# Vertebral Compression Fractures in Very Early Onset Inflammatory Bowel Disease

**DOI:** 10.1097/PG9.0000000000000283

**Published:** 2023-01-19

**Authors:** Lauren V. Collen, Scott B. Snapper, Rebecca J. Gordon

**Affiliations:** From the * Division of Gastroenterology, Hepatology, and Nutrition, Boston Children’s Hospital, Harvard Medical School, Boston, MA; † Division of Gastroenterology, Brigham and Women’s Hospital, Harvard Medical School, Boston, MA; ‡ Division of Endocrinology, Boston Children’s Hospital, Harvard Medical School, Boston, MA.

**Keywords:** bone health, Crohn’s disease, monogenic, osteoporosis, pediatrics

## Abstract

To describe clinical characteristics and outcomes of 3 patients with very early onset inflammatory bowel disease (VEOIBD) and vertebral compression fractures.

**Methods:**

Patients with VEOIBD receiving care at a single tertiary center were prospectively enrolled in a longitudinal data repository. Retrospective chart review was performed to identify clinical characteristics and comorbidities. Those with clinically apparent vertebral compression fractures subsequently underwent an additional chart review focused on bone health.

**Results:**

Three out of 216 (1.4%) patients with VEOIBD had symptomatic vertebral compression fractures. Of the 3 patients with vertebral compression fractures, all had Crohn’s disease, 2 had monogenic inflammatory bowel disease, and all reported back pain. One patient notably had a normal dual-energy X-ray absorptiometry, highlighting a potential limitation of dual-energy X-ray absorptiometry to identify increased skeletal fragility in this population. Risk factors for suboptimal bone health included chronic inflammation secondary to poorly controlled inflammatory bowel disease, substantial glucocorticoid exposure, chronic use of other medications associated with suboptimal bone health including proton pump inhibitors and granulocyte colony-stimulating factor, and solid organ transplant. Patients treated with bisphosphonates had improved clinical outcomes, with resolution of back pain and increased bone mineral density.

**Conclusions:**

Vertebral compression fracture should be considered in the differential diagnosis of patients with VEOIBD and back pain, especially in those with other risk factors for suboptimal bone health. Treatment of compression fractures with bisphosphonates resulted in resolution of back pain and improved bone density.

**What Is Known?**

- Pediatric patients with inflammatory bowel disease are at risk for suboptimal bone health.

**What Is New?**

- We found symptomatic vertebral compression fractures in 1.4% of a large single-center cohort of patients with very early onset inflammatory bowel disease (VEOIBD).
- Patients with VEOIBD and compression fractures presented with back pain and had multiple risk factors for suboptimal bone health, especially poorly controlled inflammatory bowel disease and substantial glucocorticoid exposure.
- Treatment of compression fractures with bisphosphonates resulted in resolution of back pain and improved bone density in patients with VEOIBD.

## INTRODUCTION

Inflammatory bowel disease (IBD), comprised of Crohn’s disease (CD) and ulcerative colitis, is characterized by chronic intestinal inflammation with a relapsing-remitting course. While the etiology of IBD is most often multifactorial, triggered by environmental factors in a genetically susceptible host, over 80 monogenic causes of IBD have been identified (1–3). Very early onset inflammatory bowel disease (VEOIBD) is defined as IBD onset before age 6 and carries higher likelihood of monogenic disease, with close to 8% of VEOIBD patients having known monogenic causes in 2 recent reports of VEOIBD patients with universal access to whole exome sequencing (4,5). VEOIBD has historically been associated with a more aggressive disease course (6,7), but recent work has demonstrated monogenic disease status, as opposed to age of disease onset, may be the more important driver of severe outcomes (4).

Low bone density (ie, increased risk of fracture) and osteoporosis are prevalent in patients with IBD. There has been increasing recognition of suboptimal bone health in pediatric patients with IBD (8,9); however, little is known about bone health in the VEOIBD population. Failure to attain peak bone mass in childhood and adolescence, as well as accelerated bone loss in adulthood, are both associated with an increased risk of fracture in adulthood (10,11). Decreased bone accrual in IBD is likely multifactorial, with factors including chronic inflammation, malnutrition, decreased gastrointestinal absorption, exposure to parenteral nutrition, lean muscle mass deficits, decreased growth factors, delayed puberty, decreased physical activity, exposure to glucocorticoids, and vitamin D deficiency potentially contributing.

Few studies have examined compression fractures (ie, vertebral fracture) in children with IBD, and to our knowledge, this is the first study to comprehensively evaluate them in VEOIBD. The finding of 1 or more compression fractures is indicative of osteoporosis (12). One prior case series described pediatric patients with CD and compression fractures, ranging in age from 10.6 to 16.8 years, all of whom had substantial glucocorticoid exposure, ileal disease, and low bone mineral density (BMD) (13). Another case report described a 7-year-old with ulcerative colitis, on glucocorticoids, with compression fractures successfully treated with pamidronate (14). In a case-control study of pediatric patients with IBD, aged 5–20 years, there was a significantly higher percentage of patients with IBD who had compression fractures compared with controls, indicative of a likely increased risk of subclinical compression fractures in pediatric IBD (15). In this report, we describe 3 patients with VEOIBD and symptomatic compression fractures.

## METHODS

Two hundred sixteen VEOIBD patients were prospectively enrolled in the Boston Children’s Hospital IBD Longitudinal Data Repository, from 2012 to 2020, per the protocol detailed in Collen et al (4). Three patients from this cohort were noted to have clinically apparent compression fractures (4). A retrospective chart review of these 3 patients was performed to obtain clinically relevant information, including dual-energy X-ray absorptiometry (DXA) measures to assess BMD and body composition, laboratory data, anthropometric data, and IBD clinical course. Laboratory and anthropometric data were collected from the visit closest to the DXA. Data regarding the IBD clinical course were from IBD diagnosis to date of DXA. DXA measurements were obtained in all participants, as part of their clinical care, via a Hologic Discovery A scanner (Hologic Inc, Bedford, MA). Skeletal outcomes extracted include bone mineral content (g) and areal BMD (g/cm^2^). Body composition, including lean and fat mass, and appendicular lean mass index (ALMI) were obtained from total body scans. Height *Z* score adjusted BMD *Z* scores of available skeletal sites were calculated as these outcomes are less confounded by short stature and bone size in pediatric patients (16,17). Image analysis was performed using software from Hologic Inc and Food and Drug Administration-approved pediatric reference data (18). Coefficient of variation of DXA measures in adolescents are ≤1.2% (19). *Z* scores for the body composition data were generated using pediatric reference data (20). BMD *Z* scores are defined as low if *Z* score is less than or equal to –2.0 and borderline low if between –1.0 and –2.0 (12).

The study was approved by the Institutional Review Board at Boston Children’s Hospital. All patients included in this study and their parents/guardians provided informed consent and assent where applicable.

## RESULTS

Three out of 216 patients with VEOIBD in this cross-sectional cohort (1.4%) had compression fractures. All 3 patients we describe had CD and 2 out of 3 had monogenic VEOIBD. Additional demographic and clinical features are summarized in Table 1.

**TABLE 1.** Demographics and clinical characteristics of patients with VEOIBD and vertebral compression fractures

	Case A	Case B	Case C
"Demographics and clinical characteristics" can be moved to column 1 title.			
Sex	Female	Female	Female
Race	Asian	White	White
Age at IBD diagnosis	4 y, 8 mo	1 y, 11 mo	2 y, 2 mo
Age at compression fracture diagnosis	6 y, 9 mo	9 y, 5 mo	10 y, 9 mo
IBD diagnosis	CD	CD	CD
IBD location	Colonic	Ileocolonic	Ileocolonic and upper tract proximal to LoT
IBD behavior	Penetrating; perianal	Nonstricturing, nonpenetrating	Stricturing
IBD maintenance therapy at compression fracture diagnosis	Azathioprine	Infliximab	Infliximab
Glucocorticoid exposure	Chronic, >12 mo	Chronic, >8 y	None
IBD surgery	Fistulectomy	Colectomy	None
Monogenic disease	CGD	None	GSD1b
Extraintestinal manifestations	None	None	Oral aphthae, pyoderma gangrenosum
Comorbidities	Recurrent abscesses, osteomyelitis, pulmonary aspergillosis	Heart transplant, chronic kidney disease, hyperparathyroidism, iatrogenic Cushing syndrome, adrenal insufficiency, psoriasis, G-tube	Chronic neutropenia, G-tube
TPN use	No	Yes	No
Consanguinity	Yes	No	Yes
Anthropometric parameters			
Weight-for-age *Z* score	–0.21	0.74	–0.96
Height-for-age *Z* score	–1.06	–1.56	–2.30
BMI-for-age *Z* score	0.57	1.71	0.56

BMI = body mass index; CD = Crohn’s disease; CGD = chronic granulomatous disease; GSD1b = glycogen storage disease type 1b; G-tube = gastrostomy tube; IBD = inflammatory bowel disease; LoT = ligament of Treitz; mo = months; TPN = total parental nutrition; VEOIBD = very early onset inflammatory bowel disease; y = years.

### Clinical History

Case A is a patient with chronic granulomatous disease (CGD) who presented with chronic diarrhea and perianal disease at age 5, prompting endoscopic evaluation and diagnosis of CD. She was initially treated with aminosalicylates, then azathioprine, but remained glucocorticoid-dependent for over 12 months. She additionally had a history of chronic proton pump inhibitor (PPI) use. At age 6, she developed back pain following a fall at a playground and was found to have a T12, and possibly T11, compression fracture. Medical attention was not sought immediately following her fall, but rather this history of trauma was elicited only later during the evaluation of back pain; thus, more detailed information about mechanism was not available. She had no prior fracture history but did have other complications of glucocorticoid dependence, including iatrogenic Cushing syndrome and adrenal insufficiency, and poor linear growth. Her compression fracture was managed with a brace and calcium supplementation. She continued taking a multivitamin but did not require additional vitamin D supplementation. Weight-bearing physical activity was encouraged. She was not treated with bisphosphonates, as her BMD was not low, and she clinically improved with conservative management. She ultimately underwent curative hematopoietic stem cell transplant at age 10 for her CGD and colitis.

Case B is a patient with history of heart transplant on day of life 6 for pulmonary atresia who developed bloody diarrhea, prompting endoscopic evaluation and diagnosis of CD at age 23 months. Her post-transplant course was further complicated by chronic kidney disease with associated secondary hyperparathyroidism, iatrogenic Cushing syndrome with adrenal insufficiency, psoriasis, and gastrostomy tube dependence. Her CD was initially treated with mesalamine but was inadequately controlled so she transitioned to infliximab at age 5. In addition to IBD-directed therapy with infliximab, she was taking tacrolimus and glucocorticoids for post-transplant immunosuppression. At age 9, she began to complain of significant back pain with no preceding trauma and was found to have compression fractures, initially L3, and 2 months later T11–L2. This was in the setting of inadequately controlled CD despite a regimen of infliximab, tacrolimus, and glucocorticoids. Following diagnosis of compression fractures, her glucocorticoids were slowly weaned, and she ultimately achieved corticosteroid-free clinical remission of her CD on methotrexate and tacrolimus. She had no other fracture history. She was treated with pamidronate and continued her calcium, cholecalciferol, and calcitriol. Serial DXA demonstrated increasing BMD, most recently with greater than expected interval gains for age at both the total body less head (TBLH) and lumbar spine.

Case C is a patient with glycogen storage disease type 1b (GSD1b) and chronic neutropenia who was diagnosed with CD at age 2. She had an initially mild CD course and was managed with aminosalicylates and optimization of neutropenia with granulocyte colony-stimulating factor. She was additionally taking PPI chronically. At age 10, she presented with bloody diarrhea and severe abdominal and back pain, limiting her ability to get out of bed for the preceding 6 weeks and prompting hospitalization for evaluation. Restaging colonoscopy revealed severe pancolitis. She was initiated on infliximab with some immediate improvement in diarrhea and abdominal pain, but she continued to complain of severe back pain with no preceding trauma. Spinal X-rays demonstrated compression fractures of T10–L1, L4 and L5. She had no prior fracture history. She had no history of glucocorticoid exposure, which was avoided due to her underlying GSD1b. She was treated with thoracic lumbar sacral orthosis bracing, zoledronic acid and cholecalciferol, with resolution of her back pain. She has continued on zoledronic acid for over 2 years, as serial DXA have shown interval improvement in her BMD at both the TBLH and hip but remain borderline low. She will continue zoledronic acid with serial reassessment by DXA every 1–2 years.

### Dual-Energy X-Ray Absorptiometry

DXA *Z* scores were low in 2 of the 3 participants assessed (Table 2). Height-adjusted *Z* scores demonstrated similar trends, with borderline low *Z* scores in 2 of the 3 participants. We typically take note of *Z* scores in the borderline low range in the setting of chronic diseases that adversely affect bone accrual, including IBD. In case A, DXA was performed soon after she was diagnosed with compression fracture and showed *Z* scores within the normal range, highlighting a limitation of DXA. In contrast, in cases B and C, DXA *Z* scores were low. The lowest *Z* scores were for TBLH in both cases B and C, which may reflect concurrent short stature disproportionally affecting the TBLH measurement over other anatomic sites.

**TABLE 2.** DXA and laboratory values

	Case A	Case B	Case C
DXA			
Age at DXA	6 y, 11 mo	10 y, 9 mo	10 y, 11 mo
Height at DXA	114 cm (11.82%)	124.6 cm (0.59%)	122.8 cm (0.19%)
TBLH *Z* score	–0.69	–3.8	–4.0
Ht-adjusted TBLH *Z* score	0.10	–1.90	–1.90
Lumbar spine *Z* score	0.70	–3.0	NA
Ht-adjusted lumbar spine *Z* score	1.17	–1.35	NA
Hip *Z* score (total hip/femoral neck)	NA	NA	–3.4/–3.7
Ht-adjusted hip *Z* score	NA	NA	–1.69
Fat kg (%)	7.844 (34.8%)	24.331 (51.9%)	15.585 (48.0%)
Appendicular lean/height^2^ (kg/m^2^) (*Z* score)	3.97*	5.27 (–0.2)	3.58 (–3.5)
Laboratory values			
Calcium (mg/dL; ref 8.0–10.5)	9.4	10.4	8.4
PTH (pg/mL; ref 10.0–65.0)	—	19.4	28.7
25-Hydroxy vitamin D (ng/mL; ref 30.0–80.0)	33.7	24.2	42.0
1,25-Dihydroxy vitamin D (pg/mL; ref 19.9–79.3)	—	—	33.9
CRP (mg/dL; ref <0.50)	0.62	2.11	7.73
ESR (mm/h; ref 0–30)	37	101	108


CRP = C-reactive protein; DXA = dual-energy X-ray absorptiometry; ESR = erythrocyte sedimentation rate; h = hours; Ht = height; mo = months; NA = not available; PTH = parathyroid hormone; ref = reference range; TBLH = total body less head; y = years.

*Unable to calculate *Z* score due to young age.--- = not available.

Abnormal body composition was found in all 3 patients, with increased fat measures and corresponding lower lean mass, although notably robust pediatric body composition reference ranges are not currently available. For case A, her fat mass, lean mass, and total mass were 7.8 kg (34.6%), 14.0 kg, and 22.6 kg, respectively. Her ALMI was 3.97 kg/m^2^, with *Z* score not available due to her young age. For case B, her fat mass, lean mass, and total mass were 24.3 kg (51.9%), 22.0 kg, and 46.9 kg. Her ALMI was 5.27 kg/m^2^ with *Z* score –0.2. For case C, her fat mass, lean mass, and total mass were 15.6 kg (48.0%), 16.1 kg, and 32.5 kg. Her ALMI was 3.58 kg/m^2^ with *Z* score –3.5.

## DISCUSSION

Osteoporosis and increased bone fragility (ie, fracture risk) are established extraintestinal manifestations of IBD (21,22). However, we are the first to report on this extraintestinal feature in the VEOIBD subpopulation, who likely have unique risk factors for suboptimal bone accrual and unique challenges in diagnosis of osteoporosis. In all 3 cases, IBD was one of multiple risk factors for poor bone health. One of the 3 patients we present (case A) had treatment refractory IBD, resulting in chronic, poorly controlled inflammation and substantial glucocorticoid exposure, which were her primary risk factors for compression fractures. She also had CGD, which has an unknown effect on bone, although other immunodeficiencies (eg, combined variable immunodeficiency, HIV) have been clearly linked with increased risk of osteoporosis (23,24). In case B, the patient had additional risk factors of solid organ transplant, substantial glucocorticoid exposure (for post-transplant immunosuppression), and chronic kidney disease with secondary hyperparathyroidism. In case C, the patient had the additional risk factors of granulocyte colony-stimulating factor exposure, which has been associated with suboptimal bone accrual, and poorly controlled GSD 1b (25,26). Notably, 2 of the 3 patients (cases A and C) had the additional risk factor of prolonged PPI usage, which has been associated with increased fracture risk in children and young adults (27,28). While vitamin D and calcium levels were measured following compression fracture diagnosis as part of their bone health evaluation, these represent only a snapshot in time and do not reflect longitudinal vitamin D status and calcium intake, which are important to bone health, highlighting a limitation of the retrospective nature of this study.

Collectively, these cases highlight that patients with VEOIBD, in particular those with additional risk factors, may be especially vulnerable to low BMD and compression fractures, as their exposures to risk factors for suboptimal bone health begin at an early age. In these cases, risk factors for suboptimal bone health beyond VEOIBD included solid organ transplant, GSD1b, and chronic exposure to glucocorticoids and other medications impacting bone health. Furthermore, VEOIBD is a heterogeneous disease group with phenotypes ranging from mild to severe (4), and it is important to take into account each individual patient’s unique risk factors and comorbidities when considering their bone health.

DXA, assessing areal BMD, is likely not fully capturing bone fragility in VEOIBD, and does not assess volumetric BMD, bone microarchitecture, bone quality, or strength. Many patients with VEOIBD have comorbid short stature and delayed puberty, both of which are known to alter BMD *Z* scores and accentuate the skeletal deficits in an additive way (ie, making the *Z* score appear artificially low) (12). Since smaller bones will have lower BMD, assessing height-adjusted BMD *Z* scores is clinically useful (12,29). In our cohort, participants’ *Z* scores improved when they were height-adjusted (Table 2). Interestingly, 1 patient with compression fractures had normal BMD. DXA assesses only BMD (ie, bone quantity and density) and does not assess bone quality or microarchitecture, which are other key contributors to bone strength. Other imaging modalities, such as peripheral quantitative computed tomography, trabecular bone score, and magnetic resonance imaging may be useful to assess bone parameters in patients with VEOIBD, as these imaging modalities are not influenced by bone size and additional characteristics, including bone quality, muscle quantity, and bone strength can be quantified.

Current recommendations for bone health monitoring in IBD include obtaining DXA at diagnosis of IBD to enable screening for low BMD and serial repeat scans in those with low BMD, severe disease, and other risk factors (30,31). The earliest age at which a DXA can be performed is typically 4 or 5 years old (depending on the DXA machine and available reference data) and *Z* scores are routinely available for ages 5 and older. DXA scans are therefore frequently performed starting at age 5 or slightly older, taking into consideration the patient’s ability to remain still for the scan. This can make timely diagnosis of low BMD and osteoporosis challenging in patients with VEOIBD. Serial DXA scans should be considered in patients with known low BMD to assess their interval bone accrual and in those with severe or refractory disease (eg, prolonged malnutrition, suboptimal growth velocity, or repeated courses of glucocorticoids). The minimum time interval between DXA scans is 1 year, but the optimal timing between interval scans is individualized based on each patient’s unique clinical course.

In patients with VEOIBD and concern for bone fragility, including both compression fractures and low BMD, there are frequently modifiable risk factors that can optimize bone health including: (1) goal 25-hydroxy vitamin D level of between 30 and 50 ng/mL to maximize calcium absorption and ensure normal parathyroid hormone in this at-risk population (32,33), with possibly higher 25-hydroxy vitamin D levels in the 40–60 ng/mL range being beneficial for immune regulation and homeostasis. It is common within the setting of IBD to require higher doses of vitamin D to achieve and maintain vitamin D sufficiency (34); (2) achieving the recommended daily allowance for age of calcium, which is: 700 mg/d in 1–3 years old, 1000 mg/d in 4–8 years old, and 1300 mg/d in 9–18 years old (35). This goal is best achieved by dietary intake of calcium-rich foods, but if unable to consume via the diet, calcium supplementation is an alternative; (3) ensuring normal range body mass index; (4) increasing weight-bearing physical activity; (5) minimizing use of other medications known, or with potential, to adversely affect bone health (ie, glucocorticoids, PPIs, hormonal contraceptives); and (6) assessing for other comorbidities that may be adversely affecting bone health (ie, other hormonal factors, such as amenorrhea or thyroid disease) (34).

In children with confirmed compression fractures and open epiphyses, standard-of-care includes optimizing calcium and vitamin D and consideration of bisphosphonate treatment on a case-by-case basis. In our cohort, 2 of the 3 patients (both of whom had abnormal DXA) were treated with bisphosphonate therapy. Bisphosphonate use is associated with improvement in back pain and improvement in vertebral body height (ie, bone remodeling). The duration of bisphosphonate usage is individualized, but factors considered in treatment planning include: DXA BMD *Z* scores and interval bone accrual; X-rays of the spine and determination of improved or stable compression fracture(s); presence of other fractures (ie, long bone fractures); age and notably if open epiphyses; and overall clinical status, including systemic steroid usage, chronic inflammation (assessed by inflammatory markers), and IBD activity.

In this cohort, compression fractures were all identified during evaluation of back pain. Importantly, compression fractures can be clinically asymptomatic, so our report of 1.4% prevalence of compression fractures in this VEOIBD cohort may be an underestimate. Several patients in our cohort had a clinical delay between onset of back pain and compression fracture diagnosis, including 1 patient whose pain had progressed to the point that she was nonambulatory. In some cases, it is possible the back pain was conflated with abdominal pain in the setting of poorly controlled IBD, especially in younger children who may have difficulty localizing pain. This may also reflect a lack of clinical awareness, given the rarity of compression fractures in children. In patients with VEOIBD, and especially in those on chronic glucocorticoids, with other comorbidities that predispose to suboptimal bone health, or with known low BMD, there should be a high index of suspicion for compression fracture in patients presenting with back pain. Initial evaluation for compression fractures should include spinal X-rays, typically 2-view anteroposterior and lateral radiographs. If imaging is concerning for compression fracture(s), there should be prompt evaluation by orthopedics and endocrinology, as disease course can be positively affected with appropriate treatment.

## CONCLUSIONS

Compression fractures were identified in 1.4% of a large single-center cohort of patients with VEOIBD. High index of suspicion for vertebral compression fractures is necessary in VEOIBD patients presenting with back pain. Treatment with bisphosphonates can result in improvements in back pain, BMD and in long-term bone remodeling.
